# Latent class analysis of inflammation and drug-induced liver injury phenotypes in older tuberculosis patients: associations with anxiety, depression, and insomnia

**DOI:** 10.3389/fpsyt.2025.1607551

**Published:** 2025-08-25

**Authors:** Dan Lei, Quanxian Liu, Qiong He, Zhongmin Fu

**Affiliations:** ^1^ Department of Nursing, Affiliated Hospital of Zunyi Medical University, Zunyi, China; ^2^ Department of Respiratory, Affiliated Hospital of Zunyi Medical University, Zunyi, China; ^3^ Department of Neurosurgery, Affiliated Hospital of Zunyi Medical University, Zunyi, China

**Keywords:** anxiety, depression, insomnia, tuberculosis, inflammation, drug-induced liver injury

## Abstract

**Introduction:**

Anxiety, depression, and insomnia are common among older patients with tuberculosis (TB), yet their associations with inflammatory responses and drug-induced liver injury (DILI) remain insufficiently explored. This study aimed to identify distinct inflammation-DILI phenotypes in older TB patients and examine differences in anxiety, depression, and insomnia across subgroups.

**Methods:**

In this cross-sectional study, 251 older TB patients were evaluated. Serum inflammatory markers and liver function indicators were collected, along with standardized assessments of anxiety, depression, and insomnia. Latent class analysis (LCA) was employed to classify inflammation-DILI phenotypes, and multinomial logistic regression was used to explore associations between subgroup characteristics and mental health outcomes.

**Results:**

Three latent subgroups were identified: (1) moderate inflammation with normal liver function (83.2%), (2) mild inflammation with abnormal liver function (5.3%), and (3) severe inflammation with normal liver function (11.5%). Compared with the moderate inflammation group, the severe inflammation group exhibited significantly higher rates of anxiety, depression, and insomnia. Alcohol consumption was a significant risk factor for severe inflammation (*P* < 0.05), while smoking was associated with mild inflammation and abnormal liver function (*P* < 0.05).

**Conclusion:**

Distinct inflammation-DILI phenotypes exist among older TB patients and are associated with varying psychological symptom burdens. Monitoring inflammatory markers, liver function, and mental health symptoms—especially insomnia, anxiety, and depression—may facilitate more personalized care in this vulnerable population.

## 1 Introduction

According to the *2023* Global Tuberculosis Report from the World Health Organization, China is one of the 30 high-burden tuberculosis (TB) countries. With the accelerating process of population aging in China, the incidence of TB among older individuals aged 65 and older is the highest (182.3/100,000) (1). TB incidence peaks among the older population. These patients also experience typical disease symptoms, severe adverse drug reactions, poor treatment adherence, and high rates of adverse treatment outcomes, making them a key population for TB prevention and control (2). Anxiety and depression are common mental health disorders among the older, and TB patients have a higher prevalence of mental health conditions compared to the general population (3). A study in China found that approximately 18.37% of TB patients experience anxiety, and 18.13% experience depression (4). Higher anxiety and depression scores were associated with a higher incidence of TB symptoms, more severe perceived consequences, and a reduced sense of control over the disease (5). In addition, older TB patients tend to have poor sleep quality, and chronic insomnia-induced immune dysregulation may exacerbate chronic infections (6).

The relationship between anxiety, depression, insomnia, and inflammation has been a research focus in recent years (7). About a quarter of patients with depression exhibit inflammation, as evidenced by elevated serum C-reactive protein (CRP) levels. However, key unresolved questions remain regarding symptom specificity and potential causal relationships. The level of CRP was associated with depression and sleep disturbances, although its relationship with anxiety remained unclear (8). Furthermore, TB treatment often involves long-term use of multiple drugs, with anti-TB medications being a major contributor to drug-related adverse effects (9). Drug-induced liver injury (DILI) refers to the pathological process caused by toxicity from anti-TB drugs or their metabolites or by allergic reactions in the liver (10, 11). DILI is a major adverse event during anti-TB treatment. Liu et al. (12) showed a potential association between DILI and anxiety, but this relationship has not been fully explored in TB patients with DILI.

To assess the extent and heterogeneity of inflammation and DILI in older TB patients, this study analyzed key biomarkers, including serum CRP, erythrocyte sedimentation rate (ESR), alanine aminotransferase (ALT), and total bilirubin (TBiL). CRP and ESR are common indicators of systemic inflammation, which can increase conditions such as infection, inflammation, and liver injury. These biomarkers help evaluate the level of inflammation in patients (13, 14). CRP, a sensitive marker of inflammation, is particularly valuable for early detection and monitoring of chronic diseases, especially in older patients, where monitoring inflammation is essential for preventing complications. On the other hand, ALT and TBiL are commonly used markers of liver function and are closely linked to DILI (15).

Latent Class Analysis (LCA) is a model-based, data-driven statistical method used to identify unobserved subgroups within a population based on patterns of observed variables (16). Unlike traditional variable-centered approaches, LCA is person-centered, meaning it groups individuals with similar characteristics rather than analyzing variables independently. In clinical research, LCA is particularly useful for uncovering heterogeneity in patient populations—such as differences in biomarker profiles, symptoms, or risk factors—that may not be apparent using standard classification methods (17). In the context of older tuberculosis patients, who often exhibit complex variations in inflammatory responses and liver function, LCA provides a valuable tool for identifying distinct phenotypes.

This study aimed to apply LCA to classify the inflammation and DILI in older TB patients and explore their relationship with anxiety, depression, and sleep quality. This analysis may help provide a basis for managing the psychological health and sleep quality of TB patients, thereby improving their overall treatment outcomes and quality of life.

## 2 Methods

### 2.1 Study design

This study was a cross-sectional investigation approved by the Ethics Committee of Zunyi Medical University Affiliated Hospital (KLL-2022-735). The study followed the principles outlined in the Declaration of Helsinki. Written informed consent was obtained from all participants.

### 2.2 Study population

A total of 251 older TB patients treated at Zunyi Medical University Affiliated Hospital from May 2022 to June 2023 were included in the study. The inclusion criteria were: (1) age ≥60 years; (2) meeting the diagnostic criteria for tuberculosis; (3) complete clinical data. (4) adequate communication and cognitive abilities to understand and complete the questionnaires independently or with minimal assistance. The exclusion criteria included: (1) concurrent malignant tumors, (2) concurrent systemic infectious diseases, (3) concurrent immune system diseases, and (4) concurrent extrapulmonary tuberculosis.

### 2.3 Data collection

Clinical Data: Upon admission, clinical data were collected through the medical records system, including age, gender, ethnicity, smoking history, alcohol consumption, and a history of diabetes.

Laboratory Indicators: At the time of admission, before treatment, data were also collected via the medical records system, including serum CRP, ESR, TBiL, and ALT levels. ESR and CRP were well-established markers of inflammation and were among the most reliable indicators of inflammatory conditions (18). TBiL and ALT levels could predict DILI during tuberculosis treatment (15).

The Hospital Anxiety and Depression Scale (HADS) was used to evaluate anxiety and depression symptoms upon admission (19). The HADS consisted of two subscales, the Anxiety subscale (HADS-A) and the Depression subscale (HADS-D), each containing seven items. Each item was scored on a 4-point Likert scale, with a total score range of 0–21. A score of 7 or less was considered to indicate no anxiety or depression, while a score above 7 suggested the presence of anxiety or depression.

Sleep quality was assessed using the Athens Insomnia Scale (AIS) upon admission (20). The AIS is a widely recognized self-reported sleep quality measure with eight items. It used a 4-point Likert scale (0–3), where 0 indicated “no problem” and 3 indicated “severe disturbance.” The total score ranged from 0 to 24, with a score of 0–4 indicating no insomnia and a score above 4 indicating the presence of insomnia.

### 2.4 Sample size

While our sample size of 251 is slightly below the 300-subject threshold recommended by some researchers (21), there is no universally accepted minimum for LCA. Prior studies have demonstrated that when classes are well-separated and class sizes are adequate, LCA models can produce stable and interpretable solutions with samples of this size. In our study, model entropy was acceptable and subgroup classification was interpretable, supporting the reliability of our findings.

### 2.5 Statistical analysis

The mean ± standard deviation (Mean ± SD) was used for normally distributed continuous data. For non-normally distributed data, the median and interquartile range [M (IQR)] were presented. Categorical data were presented as frequencies and percentages (n, %). LCA was performed using Mplus 8.7 software, starting with a single class and progressively increasing the number of classes. The model fit indices, including Akaike Information Criterion (AIC), Bayesian Information Criterion (BIC), sample-corrected BIC (aBIC), and entropy values, were evaluated. The Bayesian Latent Class Indicator (BLRT) and the Lo-Mendell-Rubin (LMR) test were used for model comparison. Each class contained at least five percent of patients (22–25). Based on the LCA results, patients were categorized into different inflammation-liver function phenotypes. Group comparisons were conducted using rank-sum tests and analysis of variance (ANOVA). Multinomial logistic regression was used to analyze factors influencing the latent classes, with all relevant factors included in the model due to the limited number of independent variables in this study.

## 3 Results

### 3.1 ESR, CRP, ALT, and TBiL levels in older tuberculosis patients

A total of 251 patients were included in the study, and the levels of ESR, CRP, ALT, and TBiL were assessed (**Table 1**).

**Table 1:** Basic information of ESR, CRP, total bilirubin, and ALT (n=251).

Variable	Mean ± SD	Median (IQR)	Min	Max
ESR (mm/h)	55.96 ± 23.43	50.17 (8.08)	2.00	120.00
CRP (mg/L)	45.84 ± 37.27	40.74 (10.77)	0.58	200.31
ALT (IU/L)	20.97 ± 14.84	18.50 (11.25)	3.00	79.00
TBIL(µmol/L)	12.48 ± 6.97	11.20 (6.89)	3.00	65.40

### 3.2 Results of latent class analysis

Latent class analysis was performed using ESR, CRP, ALT, and TBiL markers. Models with 1 to 6 classes were fitted, and the model fit indices are presented in **Table 2**. Based on the p-value of the LMR test, the 4-class, 5-class, and 6-class models were excluded. When the number of classes was set to three, the AIC, BIC, and aBIC values were minimized, and the LMR and BLRT p-values were less than 0.001 with an entropy greater than 0.9, indicating that the three-class model was the most appropriate. The distribution rates of the three-class model were 83.2%, 5.3%, and 11.5%, respectively (**Table 2**).

**Table 2:** Latent class analysis model fit information.

Model	K	Log (L)	AIC	BIC	aBIC	Entropy	LMR (p)	BLRT (p)	Class probabilities
C1	8	-4297.042	8610.083	8638.287	8612.926				
C2	13	-4203.732	8433.464	8479.295	8438.083	0.976	<0.001	<0.001	88.4/11.6
C3	18	-4160.056	8356.112	8419.57	8362.508	0.977	<0.001	<0.001	83.2/5.3/11.5
C4	23	-4127.74	8301.483	8382.569	8309.656	0.974	0.0799	<0.001	81.7/9.5/5.7/3.1
C5	28	-4103.936	8263.872	8362.585	8273.821	0.957	0.3811	<0.001	5.1/73.7/7.6/3.1/10.5
C6	33	-4078.348	8222.696	8339.036	8234.422	0.969	0.5989	<0.001	67.0/7.1/12.1/3.6/8.6/1.6

### 3.3 Characteristics of the latent classes

**Figure 1:**
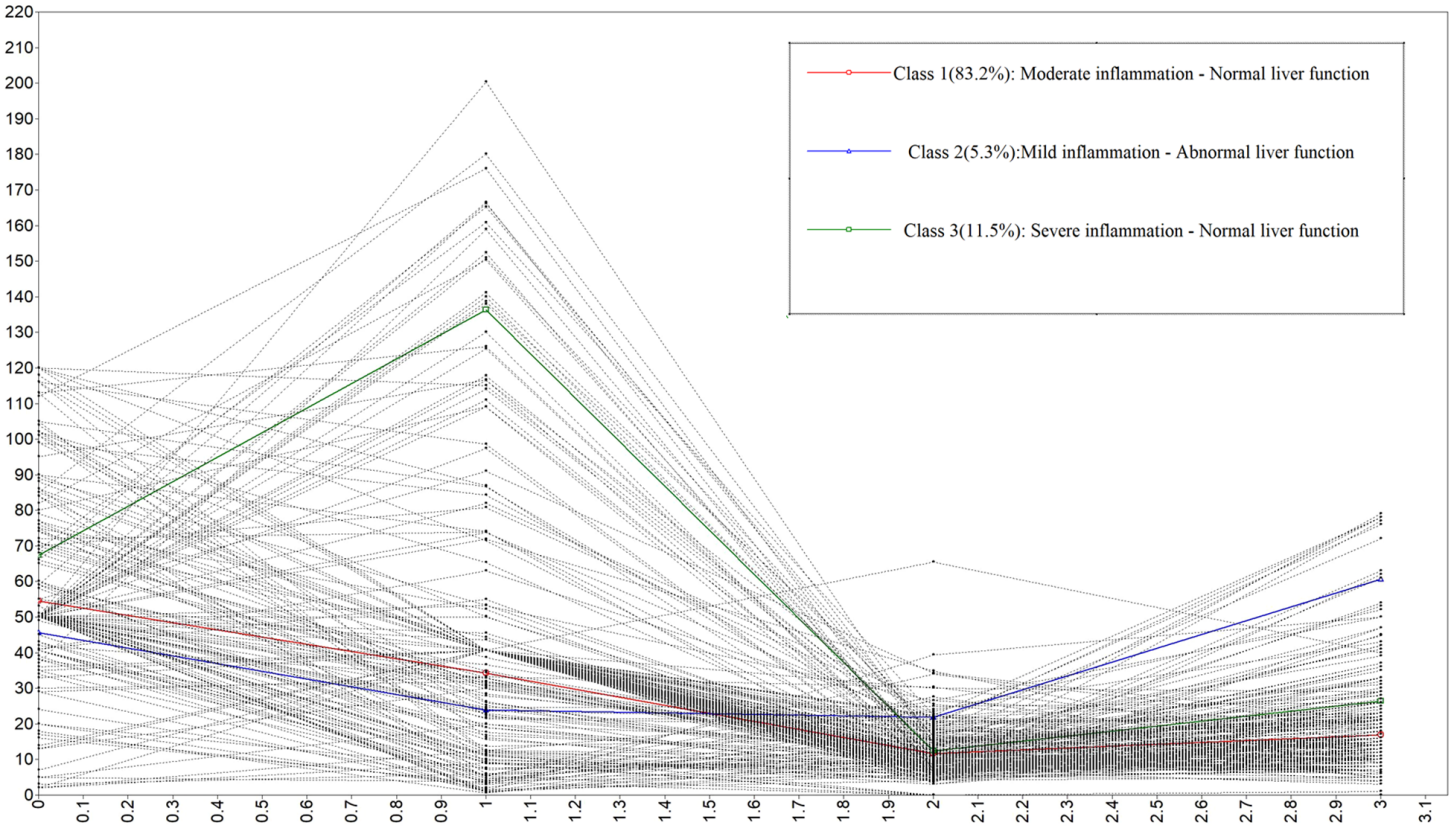
Latent class analysis of older tuberculosis patients.

The distribution of ESR, CRP, ALT, and TBiL among the different subgroups is shown in **Figure 1**. **Table 3** shows general information in three classes.

**Table 3:** General information in classes.

Variable	Class 1 N=211	Class 2 N=13	Class 3 N=27	F/Z	P-Value
Age (Mean ± SD)	70.70 ± 6.09	70.62 ± 8.69	69.81 ± 5.22	0.246	0.782
Gender n(%)					
Female	135 (64.0)	8 (61.5)	20 (74.1)	1.137	0.598
Male	76 (36.0)	5 (38.5)	7 (25.9)		
Nationality n(%)					
Non-Han	189 (89.6)	12 (92.3)	22 (81.5)	1.779	0.397
Han	22 (10.4)	1 (7.7)	5 (18.5)		
Smoker n(%)					
Yes	80 (37.9)	7 (53.8)	13 (48.1)	2.168	0.338
No	131 (62.1)	6 (46.2)	14 (51.9)		
Drinker n(%)					
Yes	60 (28.4)	4 (30.8)	13 (48.1)	4.279	0.107
No	151 (71.6)	9 (69.2)	14 (51.9)		
TB History n(%)					
New TB	190 (90.1)	12 (92.3)	27 (100.0)	1.958	0.386
Recurrence	21 (9.9)	1 (7.7)	0 (0.0)		
Diabetes n(%)					
Yes	16 (7.6)	1 (7.7)	3 (11.1)	0.813	0.692
No	195 (92.4)	12 (92.3)	24 (88.9)		
Depression n(%)					
Yes	106 (50.2)	5 (38.5)	21 (77.8)	8.380	0.015
No	105 (49.8)	8 (61.5)	6 (22.2)		
Insomnia n(%)					
Yes	191 (90.5)	12 (92.3)	17 (63.0)	17.068	0.001
No	20 (9.5)	1 (7.7)	10 (37.0)		
Anxiety n(%)					
Yes	194 (91.9)	12 (92.3)	15 (55.6)	30.353	<0.001
No	17 (8.1)	1 (7.7)	12 (44.4)		

Class 1 (83.2% of patients, n=211): The mean levels of ESR, CRP, ALT, and TBIL were 54.86, 34.69, 11.64, and 16.95, respectively. The ESR and CRP levels were between those of Classes 2 and 3, while ALT and TBIL were within normal ranges. This class was labeled as Moderate Inflammation - Normal Liver Function. Class 1 patients represented the majority and exhibited stable biomarker levels, indicating a generally favorable prognosis requiring standard monitoring.

Class 2 (5.3% of patients, n=13): The mean levels of ESR, CRP, ALT, and TBIL were 45.32, 24.19, 22.02, and 61.46, respectively. ESR and CRP levels were lower than those in Classes 1 and 3, while ALT and TBIL were abnormal. This class was labeled as Mild Inflammation - Abnormal Liver Function. Class 2 showed elevated ALT and TBIL, suggesting potential risk for drug-induced liver injury and the need for enhanced hepatic function surveillance.

Class 3 (11.5% of patients, n=27): The mean levels of ESR, CRP, ALT, and TBIL were 64.25, 141.37, 12.39, and 26.80, respectively. ESR and CRP levels were the highest, while ALT and TBIL were within normal ranges. This class was labeled as Severe Inflammation - Normal Liver Function. Class 3 is characterized by markedly elevated CRP and ESR, alongside the highest levels of anxiety, depression, and insomnia, highlighting the importance of early psychological intervention and close follow-up. This phenotype-based classification may aid in tailoring follow-up and supportive care strategies in older TB patients.

### 3.4 Multivariate analysis of factors influencing the latent classes in older tuberculosis patients

Compared to Class 1, the risk factors for Class 3 included being a drinker, experiencing anxiety, insomnia, and depression (*P*<0.05). Compared to Class 3, smoking was a risk factor for Class 2, while being a drinker and having depression were more likely to be associated with Class 3 (*P*<0.05; **Table 4**).

**Table 4:** Logistic analysis of factors influencing the latent classes in older tuberculosis patients.

Class^α^	Variable	B	SE	p-value	OR	95%CI	
						Lower	Upper
1.00	Age	0.019	0.044	0.662	1.019	0.936	1.111
	Male	0.259	0.666	0.697	1.296	0.351	4.777
	Han Ethnicity	0.794	0.649	0.221	2.212	0.620	7.896
	Smoker	0.922	0.820	0.261	2.514	0.504	12.534
	Drinker	-1.769	0.813	0.030	0.171	0.035	0.840
	New TB	0.791	0.813	0.331	2.206	0.448	10.867
	Diabetes	-0.815	0.810	0.314	0.443	0.091	2.164
	Depression	-1.194	0.538	0.027	0.303	0.105	0.871
	Insomnia	-1.508	0.549	0.006	0.221	0.075	0.649
	Anxiety	-2.052	0.518	<0.001	0.128	0.047	0.354
2.00	Age	0.020	0.063	0.747	1.021	0.901	1.156
	Male	-0.677	1.100	0.539	0.508	0.059	4.392
	Han Ethnicity	1.135	1.243	0.361	3.112	0.272	35.548
	Smoker	2.506	1.217	0.040	12.257	1.128	133.201
	Drinker	-2.260	1.114	0.043	0.104	0.012	0.926
	New TB	0.762	1.318	0.563	2.142	0.162	28.377
	Diabetes	-0.871	1.330	0.512	0.418	0.031	5.675
	Depression	-1.785	0.791	0.024	0.168	0.036	0.791
	Insomnia	-1.518	1.187	0.201	0.219	0.021	2.245
	Anxiety	-1.984	1.167	0.089	0.137	0.014	1.353

α: class 3 is the reference category.

## 4 Discussion

We identified three distinct classifications of inflammation and drug-induced liver injury in older pulmonary tuberculosis patients: moderate inflammation - normal liver function, mild inflammation - abnormal liver function, and severe inflammation - normal liver function. The levels of CRP and ESR in older TB patients were found to be higher than normal values, indicating the presence of varying degrees of inflammatory responses in these patients. CRP and ESR are commonly used markers of systemic inflammation, and their elevation typically signals acute or chronic inflammation. An increase in CRP reflects the activation of an acute inflammatory response, while an elevated ESR is often observed in chronic conditions or prolonged inflammation processes (13, 14). Therefore, the elevated CRP and ESR suggest that older TB patients were not only facing inflammation due to the TB infection but might also experience other systemic inflammatory responses or complications, which warranted particular attention from clinicians. A small subset of patients (n=13) exhibited mild inflammation accompanied by DILI. Drug-induced liver injury is a common complication during TB treatment, especially when using anti-tuberculosis drugs, which can cause liver damage (26). Clinicians should closely monitor liver function in older TB patients and consider the inflammatory status when making decisions regarding medication use.

Compared to the moderate inflammation group, the severe inflammation group had a higher prevalence of drinking, and individuals in this group were more prone to anxiety, insomnia, and depression. Consistent with a previous study, TB patients with higher depression scores exhibited elevated CRP levels (27). TB infection triggers the production of various cytokines (28). Cellular, neural, and humoral pathways allow these cytokines to reach the brain, where they can be excessively produced during inflammatory states. Inflammatory mediators, such as tumor necrosis factor-alpha (TNF-α) and interleukin-6 (IL-6), can reach brain tissue and influence the development of depression and sleep disorders by modulating synaptic transmission, neuronal excitability, neuronal survival, and synaptic plasticity (29, 30). Moreover, difficulties in falling asleep are common symptoms of anxiety and depression, with individuals suffering from these psychological conditions often experiencing sleep-onset insomnia. Polysomnographic data demonstrated persistent disruptions in sleep patterns in individuals with depression (31). Alcohol consumption may exacerbate inflammatory responses and is closely linked to mental health (32). Alcohol not only directly impacts the immune system but also potentially worsens inflammation through mechanisms such as altering neuroendocrine function and increasing the secretion of stress hormones while also influencing the nervous system, which can trigger or worsen depressive symptoms (33). Therefore, future interventions for TB patients should include targeted psychological support and health education to improve treatment adherence and quality of life.

Notably, there was no statistical significance in anxiety and insomnia between the severe inflammation group and the mild inflammation group with DILI. A potential explanation is that patients with DILI may be more affected by drug treatments or underlying conditions (12), making them more susceptible to anxiety. Therefore, despite the lower inflammation level in the mild inflammation group, the incidence of anxiety and insomnia was similar to that of the severe inflammation group, suggesting that the relationship between inflammation severity and psychological symptoms is not entirely linear. Additionally, we found that smoking was more prevalent among patients with mild inflammation and DILI. However, the relationship between smoking and DILI remains controversial. Wang et al. (34) showed no significant difference in smoking habits between DILI patients and controls receiving anti-TB drugs, while Camila et al. (35) found a reduced risk of anti-TB drug-induced hepatitis in active smokers compared to non-smokers, potentially due to nicotine-activating cholinergic anti-inflammatory pathways and reducing liver damage through heme oxygenase-1 induction (35). This study suggests that smoking could be a potential risk factor for DILI in anti-TB treatment. The components of cigarette smoke, including polycyclic aromatic hydrocarbons, induced various drug-metabolizing enzymes, potentially interfering with drug clearance (36). Clinicians should therefore pay particular attention to the smoking habits of older TB patients to reduce the risk of DILI.

These findings highlight an important public health implication: the need to systematically integrate psychological assessments into TB treatment protocols. Older patients with TB are not only biologically vulnerable due to inflammation and drug side effects but are also at increased risk of mental health conditions such as anxiety, depression, and insomnia (27). Routine psychological screening could help identify high-risk individuals early, promote timely intervention, and reduce the risk of poor adherence, treatment failure, and relapse. From a broader perspective, incorporating mental health care into TB control strategies aligns with the people-centered care model and may ultimately contribute to improved treatment outcomes, reduced disease burden, and enhanced quality of life at the population level.

Although this study revealed the relationship between different levels of DILI and anxiety, depression, and sleep disturbances in older TB patients, several limitations remain. Firstly, the cross-sectional design, while identifying correlations between inflammation status, psychological symptoms, and sleep disorders, cannot establish causality. Complex bidirectional or multilateral interactions may exist between inflammation, DILI, and mental health issues, which a cross-sectional design cannot effectively capture. Secondly, although validated self-report scales were used to assess anxiety, depression, and sleep disturbances, the subjective nature of these symptoms could introduce bias into the results. Thirdly, the potential influence of anti-TB drugs and hepatoprotective agents on the study outcomes represents a notable limitation, which may affect the accuracy and generalizability of our findings. Additionally, the small sample size in Class 2 may still affect the reliability of the statistical results. Therefore, future studies should increase the sample size to enhance the robustness of the statistical analysis and the generalizability of the results. Finally, the variability in treatment regimens or medications across patients could influence both their psychological states and liver function markers. Therefore, future research should adopt a prospective design, control for additional confounding factors, and include long-term follow-up to further validate these findings and explore deeper causal relationships.

## 5 Conclusion

This study identified three classifications of DILI in older TB patients using LCA. We found that patients with severe inflammation exhibited higher rates of psychological health issues, such as anxiety, depression, and insomnia. Long-term smoking may be an important risk factor for anti-tuberculosis DILI, while chronic alcohol consumption could contribute to severe inflammation. These findings highlight the need for clinicians to pay attention to the psychological health of TB patients and consider smoking and drinking habits when developing treatment plans.

## Data Availability

The datasets used and analyzed during this study are available on request from the corresponding author.

## Glossary

TB: tuberculosis
DILI: drug-induced liver injury
CRP: C-reactive protein
ESR: erythrocyte sedimentation rate
ALT: alanine aminotransferase
TBiL: total bilirubin
HADS: Hospital Anxiety and Depression Scale
AIS: Athens Insomnia Scale
LCA: latent class analysis
SD: standard deviation
M: median
IQR: interquartile range
AIC: Akaike Information Criterion
BIC: Bayesian Information Criterion
BLRT: Bayesian Latent Class Indicator
LMR: Lo-Mendell-Rubin
ANOVA: analysis of variance
